# Plant Volatile Organic Compounds: Revealing the Hidden Interactions

**DOI:** 10.3390/plants14040507

**Published:** 2025-02-07

**Authors:** Farhat Abbas, Hui-Cong Wang

**Affiliations:** 1 Key Laboratory of Biology and Genetic Improvement of Horticultural Crops-South China, Guangdong Litchi Engineering Research Center, College of Horticulture, South China Agricultural University, Guangzhou 510642, China; 2 Key Laboratory of Genetic Resources Evaluation and Utilization of Tropical Fruits and Vegetables (Co-Construction by Ministry and Province), Institute of Tropical Fruit Trees, Hainan Academy of Agricultural Sciences, Ministry of Agriculture and Rural Affairs, Haikou 571100, China; 3 Key Laboratory of Tropical Fruit Tree Biology of Hainan Province, Hainan Academy of Agricultural Sciences, Haikou 571100, China

Volatile organic compounds (VOCs), classified as secondary or specialized metabolites, are essential for plant health [1]. Plant volatiles are intricate, multi-faceted signals frequently utilized by pollinators alongside other cues, such as color. Although the entire plant, from roots to seeds to stems to leaves and fruits, produces VOCs, the flowers release the most significant quantity and number of these compounds [2,3,4]. Scent is a powerful tool for floral visitors. They can use it to gauge the quantity of reward in flowers, identify which flowers to visit, or send signals chemically similar to those pollinating insects employed in other environments [5,6]. Plants emit VOCs subterraneously to sense their surrounding community, strategize for or evade competition with adjoining flora, and serve as alert signals to proximate plants under specific circumstances. Furthermore, climate change (e.g., increased temperatures, drought stress, raised CO_2_, and O_3_) has significantly affected plant quality and the interactions between plants and their environment, both subterranean and aerial. There is still much mystery surrounding the functions of these compounds in plant interactions, biotic stress, and abiotic stress. Several reviews and research articles in this Special Issue focused on the function of plant volatiles in different plant and human lifecycles.

Herein, Abbas et al. [7] emphasize the significance of aroma compounds in horticulture crops. Floral and fruit emissions of aromas attract pollinators and humans who partake in the consumption of the fruit. Horticultural crops are primarily selected according to human preferences, making it essential to determine VOCs that align with sensory preferences to satisfy consumer demands. The authors look into the vital role played by fragrance volatile compounds in shaping the flavor and aroma profiles of horticultural crops while also discussing the industrial applications of plant-derived volatile terpenoids, especially in the food and beverage, pharmaceutical, cosmetic, and biofuel sectors. The discussion includes the methodological obstacles and challenges that hinder the progression from gene selection to host organisms and from laboratories to practical applications, as well as the possibilities for genetic engineering to enhance the commercial production of volatile terpenoids.

In another study, Afzal et al. [8] highlight the implications of heavy metal contamination on soil nitrate alteration and rice volatile organic compounds across various water management practices. The authors review current research concerning the negative impacts of cadmium on soil processes associated with the nitrogen cycle and the quality of rice, specifically its aroma, under various water management practices. The authors reported that cadmium reduces nitrification and denitrification processes under continuous flooding (CF) and alternate wetting and drying (AWD) conditions. The research indicates that AWD practices should be avoided in fields contaminated with Cd to minimize deposition and maintain excellent rice quality. The authors emphasize that a viable option is to utilize agricultural biotechnology and rhizospheric development to mitigate the translocation of heavy metals from the soil to the edible parts of plants.

Liu et al. [9] experimented on *Lilium* ‘Siberia’ and elucidated the light modulation of LoCOP1 and its function in floral scent production. The authors isolated the COP1 gene (LoCOP1) from the petals of *Lilium* ‘Siberia’ and examined its function, discovering that this protein exhibited the highest similarity to the COP1 of *Apostasia shenzhenica*. Furthermore, it was identified as a crucial nuclear factor that negatively regulates the generation and release of floral fragrance via virus-induced gene silencing (VIGS) application. The Y2H, β-galactosidase, and BiFC experiments demonstrated that LoCOP1 interacts with LoMYB1 and LoMYB3. The results showed that to regulate its breakdown in the dark using the 26S proteasome, LoCOP1 may ubiquitinate LoMYBs.

Yue et al. [10] identified and characterized twelve SABATH methyltransferase genes within the genome of *Hedychium coronarium*, elucidating their function in the biosynthesis of floral methyl jasmonate. Methyl jasmonate (MeJA) has been identified as a volatile compound in the blooming flowers of *H*. *coronarium*. *HcSABATH* expression analysis revealed that HcJMT1 arose as the primary candidate gene for the biosynthesis of floral MeJA. In vitro enzyme assays demonstrated that *HcJMT1* can catalyze the conversion of jasmonic acid to MeJA. The labella and lateral petals, where MeJA emission occurs, had the highest HcJMT1 gene expression. The two MeJA isomers, the HcJMT1 protein’s main isomers, were released post-anthesis when the HcJMT1 expression was high.

Bao et al. [11] identified an OsbZIP60-like transcription factor, which modulates the OsP5CS1 gene and the biosynthesis of 2-acetyl-1-pyrroline (2-AP) in aromatic rice. 2-AP is the primary volatile compound responsible for the aroma of fragrant rice. The authors constructed Osp5cs1 knockout mutant lines and OsP5CS1 over-expression lines through the genetic transformation of the Indica rice cultivar ‘Zhonghua11’, which involves the knockout of OsBADH2 to induce fragrance in aromatic rice. The OsbZIP60-like transcription factor positively regulates *OsP5CS1* (key gene in the 2-AP biosynthesis pathway). In short, the OsbZIP60-like transcription factor facilitated the accumulation of 2-AP.

A study conducted by Sheshukova et al. [12] demonstrated that the methanol-inducible gene (MIG) 21 in *Nicotiana benthamiana* encodes a nucleolus-localized protein that enhances viral intercellular transport and downregulates nuclear import. Gaseous methanol from the damaged plant helps neighboring healthy plants resist bacterial pathogens, but it promotes viral infections. Most methanol-inducible genes (MIGs) in *N*. *benthamiana* are linked to plant defense and intercellular transport. The authors describe NbMIG21, which possesses a nucleolus localization signal (NoLS). Colocalization studies using fibrillarin and coilin, markers for the nucleolus and Cajal bodies, demonstrated that NbMIG21p is localized within these subnuclear organelles. The enhanced expression of NbMIG21 promotes intercellular transit and replication of tobacco mosaic virus (TMV). The NbMIG21 promoter (PrMIG21) showed sensitivity to methanol, indicating that the accumulation of NbMIG21 mRNA is induced at the transcriptional level.

Peng et al. [13] implemented bibliometric methods, analyzing data from the Web of Science Core Collection from 1987 to 2022 to provide a numerical evaluation of the published literature on the floral scent. This assessment includes an examination of annual publication outputs, prominent research domains, temporal keyword trends, the geographic distribution of studies, institutional affiliations, co-organizations, and pertinent authors. A significant increase in floral aroma publications was observed, particularly in the fields of Food Science Technology, Plant Sciences, Chemistry, Agriculture, Biochemistry, and Molecular Biology. These study tendencies indicate a shift from micro-level investigations of individual pollination ecological activities of floral scent (FS) to a macro perspective highlighting FS’s overall influence on biodiversity and ecosystem stability. This transition encompasses evaluating the individual sensory characteristics of FS to a comprehensive assessment of their contribution to food production, quality, and improvements in yield.

In short, this Special Issue aims to compile articles that emphasize the significance of floral volatiles in plant longevity and human interactions. The manuscripts collated within this Special Issue emphasize the significance of volatile compounds in attracting pollinators, facilitating plant reproduction, influencing evolution, responding to internal and environmental factors, and aiding in seed dispersal. We addressed the application of contemporary analyzing computation approaches for the identification, characterization, and functional validation of post-transcriptional regulators. Numerous researchers have thoroughly examined the physiological, molecular, and biochemical signals related to plant secondary metabolites.
